# Nanostructured Chemiresistive Gas Sensors for Medical Applications

**DOI:** 10.3390/s19030462

**Published:** 2019-01-23

**Authors:** Noushin Nasiri, Christian Clarke

**Affiliations:** 1 School of Engineering, Faculty of Science and Engineering, Macquarie University, Sydney, NSW 2109, Australia; 2 Institute for Biomedical Materials and Devices, Faculty of Science, University of Technology Sydney, Sydney, NSW 2007, Australia; Christian.J.Clarke@student.uts.edu.au

**Keywords:** chemiresistive sensors, exhaled breath, diagnosis of diseases, metal-oxide Semiconductors, nanostructured devices

## Abstract

Treating diseases at their earliest stages significantly increases the chance of survival while decreasing the cost of treatment. Therefore, compared to traditional blood testing methods it is the goal of medical diagnostics to deliver a technique that can rapidly predict and if required non-invasively monitor illnesses such as lung cancer, diabetes, melanoma and breast cancer at their very earliest stages, when the chance of recovery is significantly higher. To date human breath analysis is a promising candidate for fulfilling this need. Here, we highlight the latest key achievements on nanostructured chemiresistive sensors for disease diagnosis by human breath with focus on the multi-scale engineering of both composition and nano-micro scale morphology. We critically assess and compare state-of-the-art devices with the intention to provide direction for the next generation of chemiresistive nanostructured sensors.

## 1. Introduction

Metal oxide semiconductor-based chemiresistive sensors have recently attracted significant attention for a wide variety of applications, including food processing [1,2], environmental monitoring [3,4], the agriculture industry [5], and medical diagnosis [6,7]. Among these applications, human disease detection through analyzing patient’s breath has attracted enormous attention in the past decade due to its key advantages over traditional diagnosis methods, including its non-invasive nature and real time analysis [6,8,9,10]. Human breath consists of oxygen, nitrogen, carbon dioxide, nitric oxide, ammonia, water vapor in addition to more than 1000 volatile trace species with concentrations ranging from several particles per trillion (ppt) to several particles per million (ppm) including ammonia (833 ppb), acetone (477 ppb), ethanol (112 ppb), acetaldehyde (22 ppb) and propanol (18 ppb) [11,12]. The concentration of endogenous compounds, including inorganic gases (e.g., NO, CO) and volatile organic compounds (VOCs) (e.g., ethane, pentane, ammonia, acetone, ethanol), can be altered in the breath of patients with specific pathologies and, thus, can be utilized as breath markers for diseases [6,13,14]. For example, acetone, H_2_S, NH_3_, NO, and toluene can be used to evaluate diabetes, halitosis, kidney malfunction, asthma, and lung cancer, respectively [15,16]. These VOCs in exhaled breath act as the target for chemiresistive sensors and with accurate detection can be used as a diagnosis tool for related diseases 

**Figure 1:**
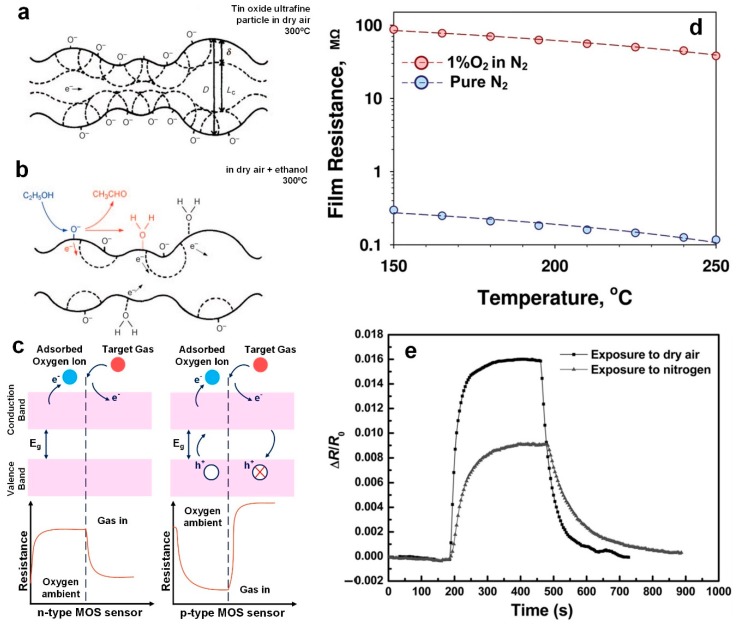
(**a**,**b**) Schematic of sensing mechanism (oxygen ions adsorption and desorption) for C_2_H_5_OH detection on the surface of tin oxide ultrafine particles in dry air and at 300 °C [1]. Reproduced with permission [1], Copyright 1982, AIP publishing. (**c**) Schematic of resistance change upon exposure to the target gas (reducing gas) for both n-type (left) and p-type (right) metal oxide semiconductor sensors. (**d**) ZnO film resistance as a function of the temperature in the absent and presence of O_2_ molecules [2] Reproduced with permission [2], Copyright 2015, Wiley Online Library. (**e**) Sensing responses of reduced graphene oxide/polyethyleneimine bi-layered sensor to 5000 ppm CO_2_ exposed to both dry air and pure nitrogen [3].

Several promising studies have been conducted on the fabrication of highly sensitive chemiresistive type exhaled breath sensors using simple and low-cost metal oxide semiconductors such as SnO_2_, MoO_3_, WO_3_, and NiO that can detect a wide variety of gases with high sensitivity [7,14,17,18,19]. The primary mechanism of metal oxide semiconductor-based gas sensors regardless of p-type or n-type is centered on reactions at the surface with the target analyte (Figure 1a,b). Initially the surface oxygen species are homogenous producing a continuous internal electron depletion and high resistance; however, when exposed to gas containing target analytes reactions at the surface neutralize these oxygen species lowering the resistance [12,20,21]. For an n-type semiconductor where the majority carrier is an electron, the adsorption of O_2_ on the surface results in electrons being trapped from the semiconductor conduction band (CB) (Figure 1a,c). This creates an electron depleted layer from the surface and within the semiconductor Debye length (δ) (Figure 1a) leading to a rise in the n-type semiconductor sensor resistance (Figure 1c). In the case of exposure to reducing gas, such as EtOH, the electrons generated from the oxidation reaction (Equation 1) are sent back to the CB resulting in a releasing of trapped electrons, increasing the electron mobility and reducing the device resistance. 

The following sensing surface reaction could be suggested for EtOH:

(1) $$C_{2}H_{5}{\mathrm{OH}}_{\left(g\right)}+\;O_{\left(\mathrm{ad}\right)}^{-}\;↔\;{\mathrm{CH}}_{3}{\mathrm{CHO}}_{\left(g\right)}+\;H_{2}O_{\left(g/\mathrm{ad}\right)}+\;e^{-}$$

where the EtOH in the gas phase, (g), reacts with the adsorbed (ad) oxygen ions on the metal oxide surface.

In contrast, the sensor’s conductivity in a p-type semiconductor is dominated by the presence of extrinsic holes. In a p-type semiconductor material, the majority charge carriers are the positive holes, resulting in an opposite effect with an increase in the resistance when it is exposed to a reducing gas [22].

In fact, the conductivity of these semiconductor-based sensors is mainly controlled by the adsorption and desorption of O_2_ molecules [12,20,21]. This can be confirmed by measuring the resistance of a ZnO film in the presence and absent of oxygen (Figure 1d). If only exposed to pure nitrogen gas, the resistance of the sensor only decreases by ~2 times when increasing the sensor temperature from 150 to 240 °C [20]. In contrast, adding the O_2_ molecules into the gas sensing system increased the film resistance by 300 times from 0.298 to 87 MΩ [20]. This indicates that the conductivity of the ZnO film is mainly controlled by adsorption and desorption of O_2_ molecules on the surface (Figure 1d). Similar results obtained by Zhou et al. [23] with their reduced graphene oxide sensor demonstrated a significantly higher sensitivity to the CO_2_ gas in air rather than in N_2_ (Figure 1e). This indicates the vital role oxygen plays in the sensing mechanism of metal oxide semiconductor-based devices.

**Figure 2:**
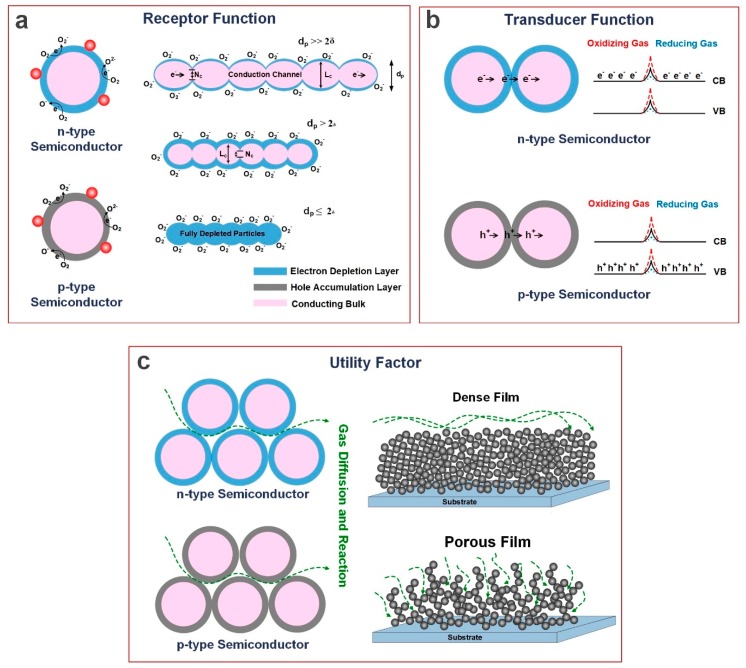
Three main factors controlling semiconductor gas sensors: (**a**) Receptor Function, (**b**) Transducer Function and (**c**) Utility Factor.

The gas adoption mechanism and consequent resistance change in a metal oxide semiconductor gas sensor mainly involves three major functions: receptor function, transducer function and utility factor (Figure 2) [24,25]. The receptor function is mainly attributed to the sensitivity and selectivity of the device and how each component responds to the surrounding environment including oxygen and the other gases [26]. In this stage, the amount of oxygen adsorbed on the surface leading to the depletion of the surface mainly governs the sensing capabilities of the device, which depends on the structure specific surface area (SSA), particularly on the particle size (*d_p_*) of the sensing structure [26]. If *d_p_* >> *2δ*, the sensing mechanism is determined by transferring electrons at the particle’s grain boundary resulting in a low sensitivity (Figure 2) [12,20]. If *d_p_* > *2δ*, a large portion of the bulk participates in the sensing mechanism leading to a moderate sensitivity (Figure 2a) [12,20]. In contrary, if *d_p_* ≤ *2δ* the entire particle is electron depleted with no mobile charge carrier, leading to significantly high resistance with very low baseline currents [12,20].

The transducer function, as the second major, is an interparticle issue related to how the surface phenomenon is transformed into a change in electrical resistance of the sensor [27], and how the response from each particle is represented by that of the whole device (Figure 2b). The chemical interaction of the semiconductor surface creates an electrical signal in the transducer function, which is mainly dominated by the surface potential and potential barriers formed between grains, trapping states in grain boundaries and defect states in the semiconductor structure [12,28,29]. Schottky barriers between two grains impede electrons transferring across the boundary [29]. Therefore, the boundaries between grains act as transducers when the resistance change by the gas adsorption is amplified [29]. Several studies have shown the importance of optimizing the intergrain boundary for enhancing the transduction of the surface response [29,30].

Lastly, the utility factor (Figure 2c) is related to the morphological structure of the metal oxide semiconductor and consequently the diffusion and reaction of target gas through the structure pores [26,31]. The utility factor determines how the sensing performance is affected by the device structure, with the film porosity as the most important parameter in achieving the highest utility factor [20]. In a very porous structure, the target gas particles penetrate into the lowest layers of film resulting in an effective resistance variation of the sensing device (Figure 2c) [20]. However, the utility factor might be meaningless for monolayer structures such as MoS_2_ as the material structure is atomically thin and the gas adsorption is not associated to the diffusion through the material [26]. For such 2D structured sensors, the utility factor is already maximized, and the highest performance of the device should be achieved by choosing proper receptors and enhancing the interaction between target gas and sensing material [26].

## 2. Doped Metal-Oxide Semiconductor Sensors

**Figure 3:**
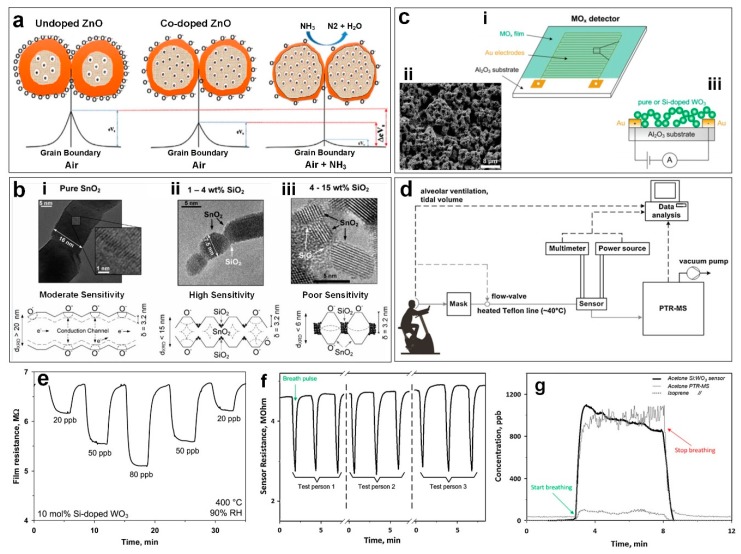
(**a**) Schematic diagram of gas sensing mechanism of undoped ZnO and co-doped ZnO thin film exposed to NH_3_ gas [36]. Reproduced with permission [36], Copyright 2015, Elsevier. (**b**) TEM images and schematic of morphological change in SnO_2_ nanoparticles (i) before and after (ii) 1–4wt.% and (iii) 4–15wt.% SiO_2_ doping [35]. Reproduced with permission [35], Copyright 2008, Wiley Online Library. (**c**) Schematic and SEM image of metal oxide sensor deposited on Al_2_O_3_ substrate with interdigitated Au electrodes [38]. (**d**) Schematic of the breath analysis experiment with the breath flow controlled by the mask and kept constant by the PTR-MS pump [14]. (**e**) 10 mol% Si-doped WO_3_ film resistance upon exposure to different concentrations of acetone from 20 to 80 ppb, at 400 ℃ and 90%RH [38]. (**f**) Si:WO_3_ sensor resistance change to short pulses of three tests conducted by different healthy volunteers with similar breath acetone concentration [14]. (**g**) Acetone concentration measured by the Si:WO_3_ sensor (thick solid line) and acetone (thin solid line) and isoprene (dotted line) concentrations measured by PTRMS during breathing of a volunteer [14]. Reproduced with permission [38], Copyright 2010, ACS Publications. Reproduced with permission [14], Copyright 2012, Elsevier.

A barrier for the implementation of metal oxide semiconductors is their slow kinetics both in response to target analytes and recovery [27]. However recent progress in the synthesis of novel nanostructures allowing for superior surface area, pore size and distribution can be used to circumvent this issue [10,13,32,33]. Several studies have reported the sensitivity enhancement of the metal oxide semiconductor-based gas sensors through processing or adding noble metal impurities [34]. By functionalizing the large surface of the sensor with catalysts such as Pt, Rh, Ag, Si and Pd many studies have shown remarkable enhancement to their kinetics [14,15,34,35]. In fact, doping enhances the sensing performance of metal oxide semiconductor-based gas sensors by modifying their micro- nanostructure and changing their activation energy and/or band gap [19,33]. Figure 3a an example of the gas sensing mechanism for doped ZnO thin films highlights how this mechanism is affected when exposed to NH_3_ gas [36].

In addition to the sensitivity enhancement and response/recovery kinetics, addition of dopants or impurities might also improve the device selectivity as each material could be selective to a specific target gas [37]. Additionally, the stability of fabricated metal oxide devices can be greatly increased by doping with other metals [35]. For instance, the thermal stability of the metal oxide semiconductor device could be improved by the solid solutions formation between metal oxide and its dopant. Tricoli et al. [35] demonstrated a SnO_2_ sensor with enhanced sensitivity and stability from optimal Si content by changing the percentage of SiO_2_ in the synthesized SnO_2_ films. Figure 3b illustrates the morphological nanostructure and sensing mechanism for the SnO_2_ sensor before and after adding SiO_2_. Initially, pure SnO_2_ sintered at 600 °C produces elongated crystals of more than seven times Debye length (*δ*) [35]. These structures (Figure 3bi) are disadvantageous for sensing as they form an un-depleted conduction channel (closed-neck morphology). During sensing, only moderate sensitivity to the target gas is expected as this morphology causes the injection of carriers to mostly affect the conductivity of the depleted region close to the surface (Figure 3bi). The second nanostructure (Figure 3bii) is formed with 1–4 wt % SiO_2_ contents causing the crystal to form small necks between the primary particles (Figure 3bii). The depleted inter-crystal boundaries of this second nanostructure enhance the injection of electrons, reducing the total resistance and giving this structure the highest sensitivity. Finally, at 15 wt % SiO_2_ contents (Figure 3biii), the large dielectric SiO_2_ regions separate the SnO_2_ crystals resulting in electrically isolated nanostructures and the poorest sensitivity [35].

In another approach, a flame-deposited portable sensor was developed using a flame spray pyrolysis (FSP) reactor. The nanostructured porous device consisted of in situ annealed Si-doped *ε*-WO_3_ nanoparticles which were deposited on the surface of water-cooled Al_2_O_3_ substrates with interdigitated electrodes (Figure 3ci and ciii) [38]. The resulting ultraporous nanostructured gas sensor (Figure 3cii), allowed rapid diffusion of the target gas as well as discharging sensing reaction products [38]. The fabricated sensor demonstrated a significantly high response to low concentrations of acetone (response of 0.1 to 20 ppb) (Figure 3e), comparable to the more complex standard methods such as selected-ion flow-tube mass spectrometry (SIFT-MS). This simple detector with its precise and sensitive detection of ultralow acetone concentrations has the potential to be used directly, for medical diagnostic applications such as diabetes monitoring/detection [38]. In addition, after flushing with humid air, the baseline is rapidly recovered, which is vital for real time applications [38].

In a similar study, Righettoni et al. [14] performed real breath measurements (Figure 3d) using a respiratory flow controlled mask connected to both Si:WO_3_ sensors and a high-sensitivity proton transfer reaction mass spectrometer (PTR-MS). Figure 3d shows the schematic of the experimental set-up during a breath analysis test. During tidal breathing the acetone and isopreene concentration in the exhaled breath of a healthy test volunteer is measured by both the Si:WO_3_ sensor (thick solid line) and PTR-MS. As presented in Figure 3f, the Si:WO_3_ resistance decreases sharply(∼3 min) and then recovers to the initial value after breath flow stopped (∼8 min) (Figure 3f). The sensor response corresponded to an acetone concentration of about 970 ppb (Figure 3g) on average at 3–8 minutes, which is well in line with the PTR-MS that measured an acetone concentration of 980 ppb (thin solid line in Figure 3g). However in addition the fabricated ultraporous Si:WO_3_ sensor had a higher signal to noise ratio compared to the PTR-MS (60 and 9, respectively) [14].

## 3. Composite Metal Oxide Semiconductor Sensors

Thanks to their high sensitivity, low fabrication cost, and long-lasting operational life, metal oxide semiconductor-based sensors have been widely researched over the past decade [6,39,40]. However, low conductivity, poor selectivity and required high operating temperature are some common shortfalls of metal oxide semiconductor devices [27,41]. Recently, graphene/metal oxide semiconductor composites have attracted significant attention as alternatives for functionalizing chemical sensors due to their high electrical conductivity and faster response dynamic. Numerous studies have reported the benefits of reduced graphene oxide (rGO)-WO_3_ nanoparticles [42], graphene-SnO_2_ nanorods [43] and graphene oxide (GO)-ZnO nanoparticles [44] in detecting various gases including NH_3_, H_2_S, and NO_2_. 

**Figure 4:**
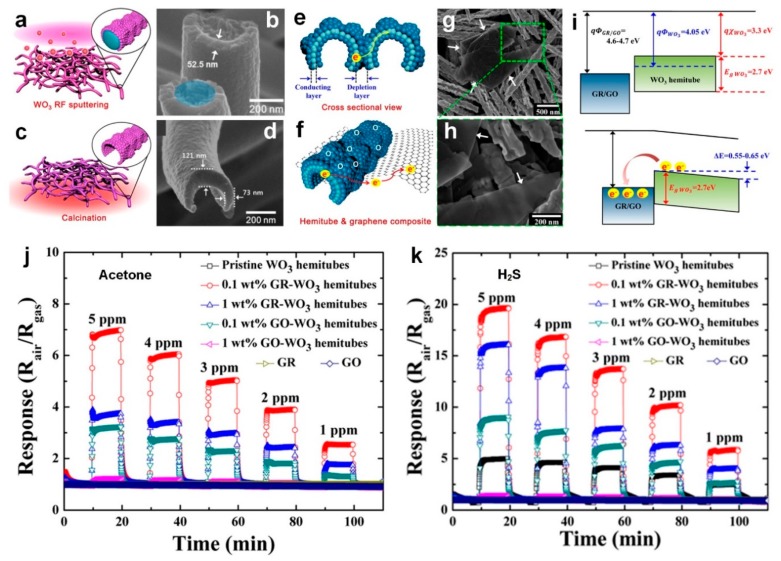
Schematic illustration and SEM images of fabricated WO_3_ hemitubes (**a**,**b**) before and (**c**,**d**) after calcination at 500 ℃ for 1 h. [4,5] (**e**) Homojunction between WO_3_ hemitubes and (**f**) a graphene-based material and a WO_3_ hemitube. SEM image of GR-WO_3_ hemitube composite at (**g**) low and (**h**) high magnification. Band structure model for GR/GO and WO_3_ hemitubes relative to the vacuum level. Sensing responses of fabricated WO_3_ hemitubes upon exposure to (**j**) acetone and (**k**) H_2_S with different concentrations from 1 to 5 ppm at 300 ℃. [4] Reproduced with permission [4], Copyright 2014, ACS Publications. Reproduced with permission [5], Copyright 2013, ACS Publications.

Recently, Choi et al. [15] reported a new nanostructured material made of WO_3_ hemitubes improved with thin graphite (GR) or GO layers. Figure 4a,c show the fabrication process for the very high surface area WO_3_ hemitubes using a nonwoven polymeric fiber template network. Initially, they synthesized the polymeric fiber template through electrospinning of a polyvinylpyrollidone (PVP)/poly(methyl methacrylate) (PMMA) composite (Figure 4a,b) [15,16]. Then, WO_3_ films where deposited onto the electrospun PVP/PMMA nanofibers by Radio Frequency (RF) sputtering method. A high temperature calcination (500 °C) resulted in decomposition of nanofibers template, and fabrication of hollow WO_3_ hemitube structures (Figure 4c,d). Lastly, a homogenous mixing method was used to functionalize the graphene-based materials onto the WO_3_ hemitubes (Figure 4f–h) [15,16].

As shown in Figure 4e, the electron depleted layers generated on the surface of pure WO_3_ hemitubes resulted in the suppressed charge transport through continuous hemitubes [15,16]. However, for the hetero-interface between WO_3_ hemitube and graphene (Figure 4f), the charge transport is significantly enhanced leading to faster response dynamics. This can be explained by the band structure of the device at the heterojunction structure, which is presented in Figure 4i. Given the respective work functions, transferring electrons from the WO_3_ hemitubes to the GR/GO becomes possible, leading to a depletion layer formation (Figure 4i, bottom) [15]. Then, upon exposure to the reducing gas, GR and GO reduce the electron concentration at the WO_3_ hemitubes surface creating larger conductivity changes for these fabricated composite sensors [15]. The sensor response, defined as (R_air_/R_gas_) is assessed at different temperatures from 200 to 350 °C with 85−95% relative humidity (RH) upon exposure to acetone (Figure 4j) and H_2_S (Figure 4k), which are well known biomarkers for diabetes and halitosis, respectively [15].

In order to detect such diseases through analyzing exhaled breath, highly sensitive detection is required as there is <1 ppm of acetone difference between healthy patients (0.9 ppm) and diabetic patients (1.8 ppm). Similarly, at least 1 ppm of H_2_S should be detected for diagnosing halitosis, which is at the edge of the minimum concentration required for humans to detect the characteristic odor from the exhaled breath of halitosis patients [15].

As presented in Figure 4j, the maximum response to acetone, 6.96 at 5 ppm, was achieved by the 0.1 wt % GR-WO_3_ sensors at 300 °C, demonstrating a 6.45-fold enhancement compared to that of single phase WO_3_ device (1.08 at 5 ppm), whereas 0.1 wt % GO-WO_3_ device demonstrated a response of 3.25 which was 3 times higher than single phase device. (Figure 4j). In fact, the WO_3_ functionalization with GR and GO has significantly enhanced the sensitivity of the metal oxide semiconductor sensor to acetone (1.08 at 5 ppm). In the case of H_2_S detection, the pure WO_3_ device showed a sensor response of 4.98 for 5 ppm of H_2_S, while functionalization with 0.1 wt % GR resulted in sensor response of 19.66 which was four times higher than pure WO_3_ device (Figure 4k). As shown graphene-based additives along with thermal aging play a significant role in the sensing properties and performance of new fabricated composite materials for the diagnosis of diabetes and halitosis by exhaled breath analysis [15].

**Figure 5:**
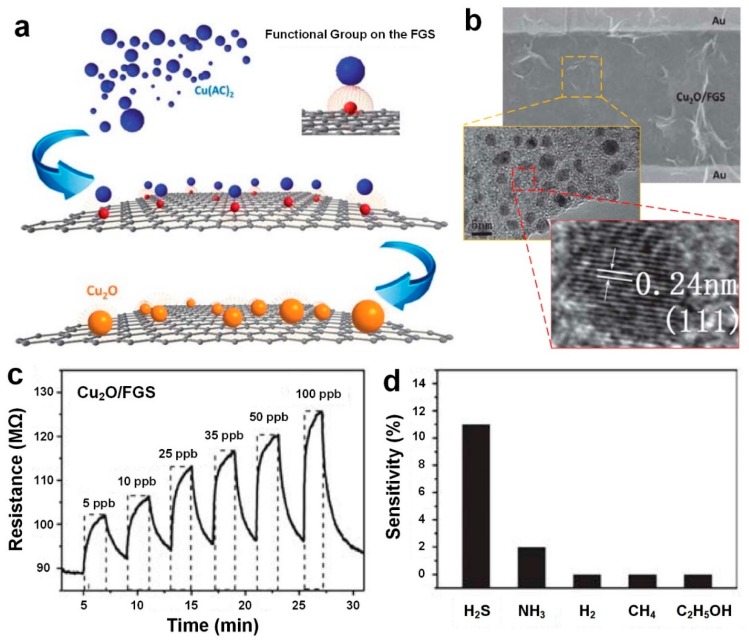
(**a**) Schematic illustration of Cu_2_O-FGS through an in-situ process. (**b**) SEM and TEM images of Cu_2_O-FGS, covering gold interdigitated electrodes. (**c**) Dynamic sensing response of Cu_2_O-FGS sensor upon exposure to H_2_S gas at different concentrations of 5 to 100 ppb. (**d**) The device sensitivity towards H_2_S (5 ppb), NH_3_ (25 ppm), H_2_ (25 ppm), CH_4_ (25 ppm) and C_2_H_5_OH (25 ppm) [6]. Reproduced with permission [6], Copyright 2013, RSC Publishing.

In another approach, Zhou et al. [45] used functionalized graphene sheets (FGS) as molecular templates to deposit a uniform and dense layer of 3 nm thick Cu_2_O nanocrystals. Figure 5a shows the schematic illustration of Cu_2_O-FGS nanocomposite fabrication, demonstrating copper acetate (Cu(Ac)_2_) precursor uniformly adsorbed onto the FGS surface [45]. First, the Cu(OH)_2_ is nucleated from Cu(Ac)_2_ at room temperature, and later is transformed to Cu_2_O under high temperature and vapor pressure (Figure 5a). During this process, the FGS function in controlling the nucleation and eliminating unfavorable aggregation [45].

The SEM image of fabricated Cu_2_O-FGS on Si/SiO_2_ substrate with gold interdigitated electrodes is presented in Figure 5b. It can be clearly seen that single Cu_2_O nanocrystals are well separated from each other with no clear aggregation of Cu_2_O nanocrystals on the FGS surface [45]. The fabricated Cu_2_O-FGS nanocomposites demonstrated higher stability against oxidation in ambient atmosphere compared to the bulk Cu_2_O device, due to their ultrafine size effect as well as interfacial effects between Cu_2_O nanocrystals and FGS. Upon exposure to H_2_S gas, the sensor demonstrated a fast, sensitive and reversible response at room temperature to significantly low concentration of gas ranging from 5 to 100 ppb as displayed in Figure 5c. This response is remarkably higher than the recent results reported in the literature so far for CuO/Cu_2_O sensors [45,46]. The Cu_2_O nanocrystals grown on the FGS provide more active sites for the adsorption of target gases. In addition, the FGS enhances transferring electrons more efficiently by acting as a conducting network, leading to a significantly high sensitivity and electron conductivity in the fabricated device [45]. Furthermore, the device selectivity was investigated upon exposure to several gases including H_2_S (5ppb), NH_3_ (25 ppm), H_2_ (25 ppm), CH_4_ (25 ppm) and C_2_H_5_OH (25 ppm) (Figure 5d). As it can be seen in Figure 5d, the device sensitivity to 25 ppm of NH_3_ gas is almost five times lower than that of 5 ppb of H_2_S, suggesting that Cu_2_O-FGS sensors have immense potential as medical diagnostic devices [45].

## 4. P-N Heterojunction Metal Oxide Semiconductor Sensors

In the past decades, several nanostructured devices have been developed to overcome the limitations of metal oxide semiconductor-based devices, including noble metal doping, surface functionalization and fabrication of core-shell structures [7,16,35,38,39]. Among them, p-n nanoscale heterojunction nanomaterials have been synthesized as great platforms for nanostructured gas sensors [7,15]. In these nanostructured devices, a nanoscale heterojunction is shaped at the core-shell boundary, resulting in a built-in electric field at the p-n interface which plays a vital role in the sensing properties of the fabricated device [39,47,48]. In fact, the built-in electric field between the nanoscale p- and n-type areas leads to the rapid separation of the charge carriers resulting in significantly faster response dynamic [39,47].

**Figure 6:**
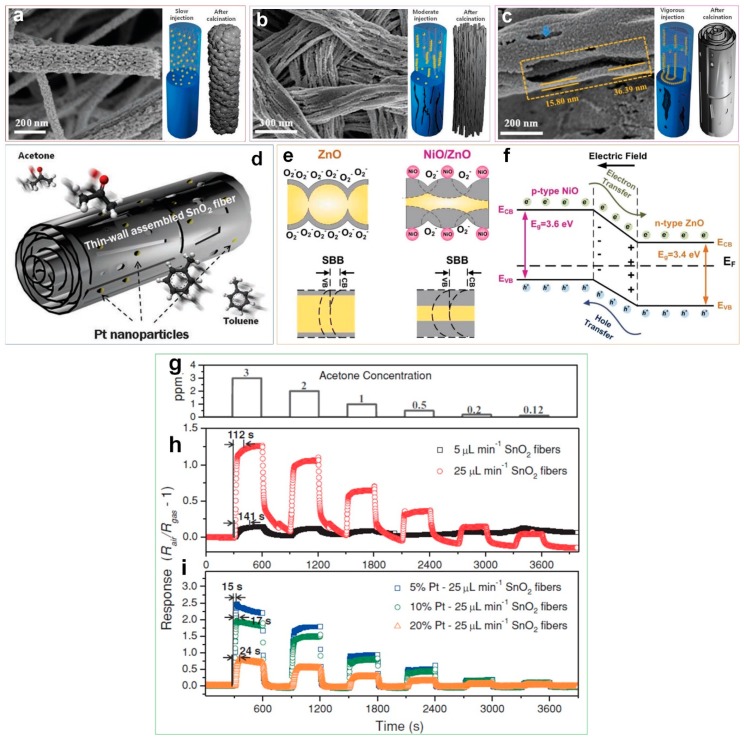
SEM images and schematic illustrations of morphological evolution of as-spun and calcined SnO_2_ fibers prepared at (**a**) 5 µL∙min^−1^, (**b**) 15 µL∙min^–1^ and (**c**) 25 µL∙min^−1^ flow rates, and (**d**) Pt-decorated thin-wall assembled SnO_2_ fibers. [50] (**e**) Schematic of photodetection mechanism for pure ZnO and NiO/ZnO heterojunction device. The grey and yellow areas represent electron depleted and conducting regions, respectively. (**f**) Schematic of electron-hole separation in the NiO/ZnO heterojunction device. [39] (**g**) The cyclic acetone response of the sensor at different concentrations, with respect to time. The acetone response of (**h**) pure SnO_2_ fibers with 5 and 25 µL∙min^−1^ flow rate and (**i**) modified fibers with 5, 10 and 20 wt.% Pt decoration [50]. Reproduced with permission [50], Copyright 2013, Wiley Online Library. Reproduced with permission [39], Copyright 2017, RSC Publishing.

Several researchers have reported the key role of heterogeneous catalyst in enhancing the sensing capability and response dynamics of semiconductor-based gas sensors [48,49,50]. Tian et al. [49] synthesized a NiO/ZnO p-n heterostructure gas sensor using a hydrothermal method, featuring a diode-like behavior able to detect ethanol concentrations of 4 to 10 ppm with fast response dynamics (6 and 22 s response and recovery time, respectively) at 200℃. In another approach, Shin et al. [50] reported the morphological evolution of hierarchical electrospun SnO_2_ fibers, composed of wrinkled thin SnO_2_ nanotubes, synthesized by microphase separation between tin and polymer precursors and changing the electrospinning flow rate (Figure 6a–c). The phase separation between the tin and polymer precursors, which are influenced by the electrospinning flow rate control (low (Figure 6a), intermediate (Figure 6b) and fast (Figure 6c)), can modify the morphologies of the SnO_2_ fibers [50]. As presented in Figure 6c, the surface of the wrinkled SnO_2_ tube’s thin-walls are extensively covered with open pores after fast flow rate electrospinning, resulting in 5 times higher gas response (6.12 at 3 ppm) compared to that of densely packed fibers (Figure 6g,h). This unique morphology featured a significant increase in the accessibility of the entire device to the target gas with all sensing layers accessible [50].

Furthermore, the fibers were functionalized by catalytic Pt nanoparticles to evaluate their sensing performance toward acetone (Figure 6d). As illustrated in Figure 6i, the response and recovery times of the Pt-decorated fibers are remarkably shortened (15 s) compared to the non Pt-decorated specimens (112 s) [50]. As presented in Figure 6d, the multilayered thin-wall assembled SnO_2_ fibers with large holes between layers allows the uniform deposition of oxidized Pt nanoparticles. This leads to an extended electron depleted region for the SnO_2_ fibers, producing nanoscale heterojunctions of PtO/SnO_2_ which are the source of the fast response and recovery speed [50]. A similar enhancement mechanism was also reported for a metal oxide semiconductor-based UV photodetector, consisting of a surface with a ultraporous network of electron depleted n-type ZnO nanoparticles coated by densely packed p-type NiO clusters which featured a 20 times faster UV response dynamic compared to the pure metal oxide device [39]. A built-in electric field created at the NiO/ZnO interface (Figure 6f) resulted in a larger upward surface band bending (SBB) (Figure 6e) and even lower dark-currents (Figure 6e) than pure ZnO [39,47]. Under UV light illumination with photon energies exceeding the ZnO band gap, photogenerated holes can rapidly migrate in the adjacent p-type domain, leading to a prolonged electron lifetime and thus increased photo-current [39,47]. Once the UV illumination is terminated, the excited electron–holes can recombine at the NiO/ZnO interface resulting in a rapid decay of the photo-current as this solid-state process does not require the re-adsorption of O_2_ molecules (Figure 6e,f) [39].

## 5. Arrays of Metal Oxide Semiconductor Sensors

As mentioned before, a major drawback for metal oxide chemiresistive sensors is the lack of selectivity to the target analyte in the presence of interfering gases. However, this problem could be tackled by arranging a group of sensors into arrays rather than using a single sensor device [10,18,51]. Chemical sensor arrays have been demonstrated to be highly effective devices to distinguish analytes [52,53]. Additionally, sensor arrays have already been applied for diagnosing, renal disease, lung cancer and diabetes [10,18,51,54]. In the sensor array model each sensor reacts distinctively to the target analyte which allows an intensity pattern matrix to be produced. This matrix can then be processed by an algorithm to identify and quantify the target analyte. This type of sensor architecture opens up the possibility for one device to be sensitive to multiple targets [18,55].

**Figure 7:**
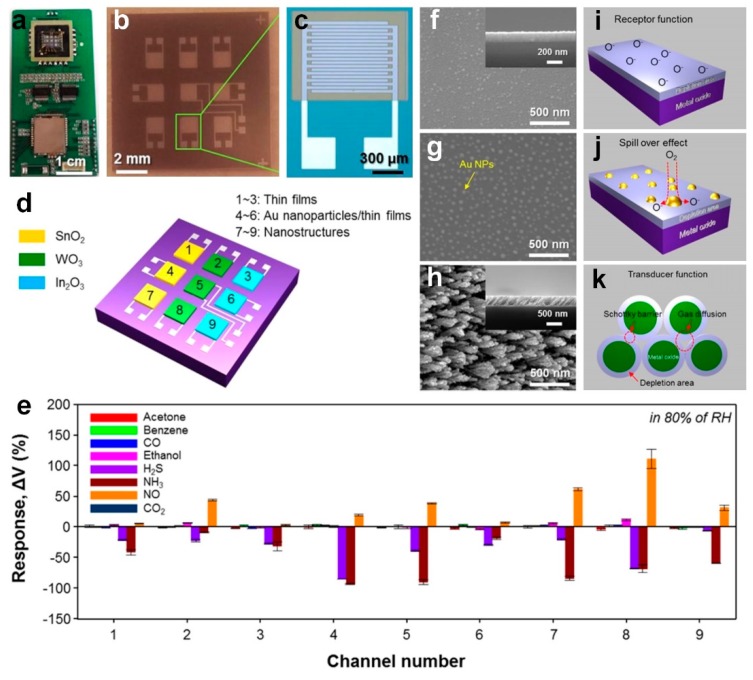

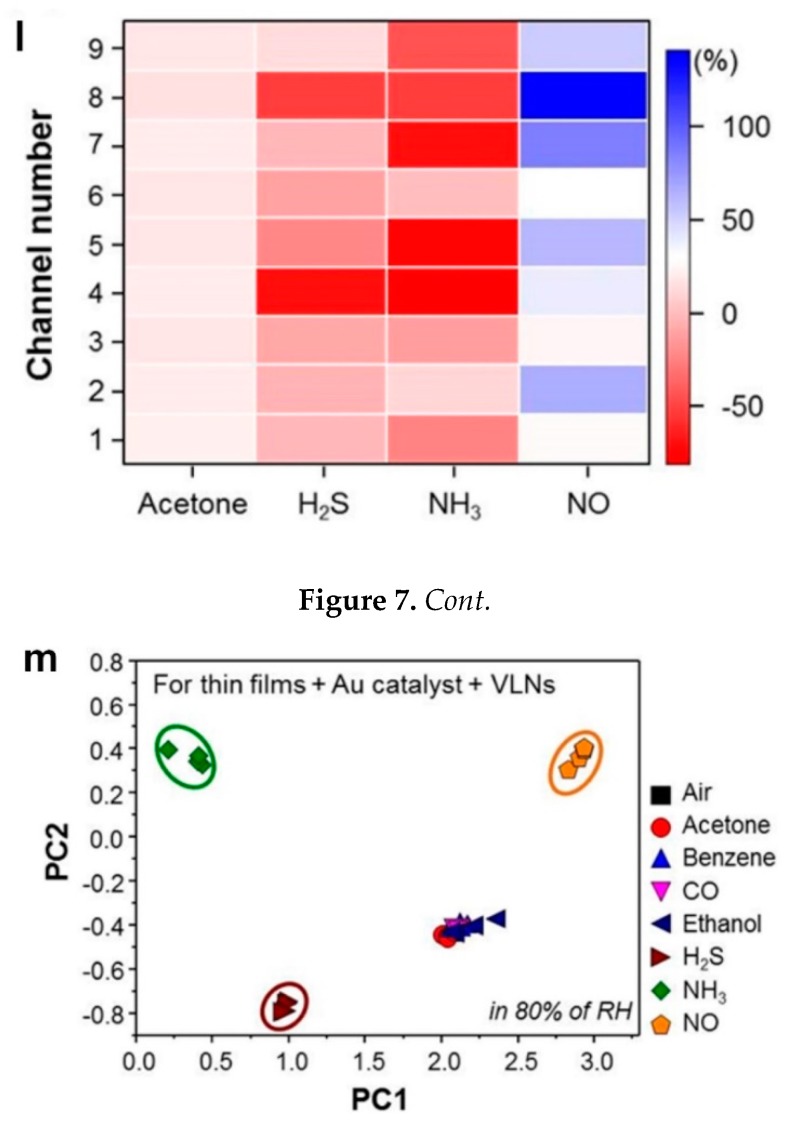
(**a**) Photograph of the signal processing circuits and the integrated chemiresistive electronic nose. (**b**) optical microscope images of the fabricated device with (**c**) a single chip containing an active layer on Pt interdigitated electrodes. (**d**) schematic illustration of the fabricated device consisting of an array of sensors. (**e**) Response patterns of the fabricated device to 8 different gases. FE-SEM images of (**f**) thin films, (**g**) Au functionalized thin films and (**h**) villi-like nanostructures with (**i–k**) the schematic illustration of sensing mechanism. (**l**) Color-coded response of the fabricated electronic nose to H_2_S, NH_3_, acetone and NO gases. (m) PCA plot showing thin films + Au functionalized thin films +VLNs responses to 8 gases in 80% RH [7]. Reproduced with permission [7], Copyright 2016, ACS Publications.

Moon et al. [18] developed an electronic nose using arrays of highly selective and sensitive metal oxide thin films, enhancing sensitive detection of several gases including NO, NH_3_ and H_2_S, in an 80% RH (similar to the composition of exhaled breath) for detection of asthma, kidney disorder and halitosis respectively. Using e-beam in a glancing angle deposition (GAD), they fabricated 3 × 3 arrays of chemiresistive sensors consisting of different nanostructured thin films (Figure 7a–d) including WO_3_, SnO_2_ and In_2_O_3_. Here, the oxygen molecules are adsorbed onto the semiconductor surface creating negatively charged species, leading to the formation of an electron depleted layer (Figure 7f,i) [18]. For the Au-functionalized thin films, a 3 nm Au film was deposited on the surface usinge-beam deposition, leading to an enhanced selectivity due to the catalytic properties of deposited nanoparticles and chemical sensitization via the spillover effect (Figure 7g,j) [18]. Furthermore, fabricated porous villi-like nanostructures (VLNs) with 37% porosity demonstrated an enhanced gas sensing through effective diffusion and adsorption of target gases (Figure 7h). This higher porosity resulted in a higher utility factor and as explained by the double Schottky barrier model an enlarged resistance variation (Figure 7k) [18].

The real-time response of each sensor at 168 ℃ and 80% RH was recorded individually to evaluate the sensing properties of the fabricated device. As presented in Figure 7e, the sensors response was significantly low for gases composed of robust bonds between carbons (including acetone, benzene and ethanol) [18]. Similarly, CO_2_ is a chemically stable gas due to its centrosymmetric structure leading to very low response from chemiresistive sensors. In contrast, the sensors response to both H_2_S (2 ppm) and NH_3_ (10 ppm) was considerably high at the relatively low temperature of 168 °C, which is attributed to the spillover effect and dissociation of the oxygen resulting in active sites [18]. The color scaled response amplitudes of the array in differentiating target gases mapped in Figure 7l highlight this selectivity [18].

**Figure 8:**
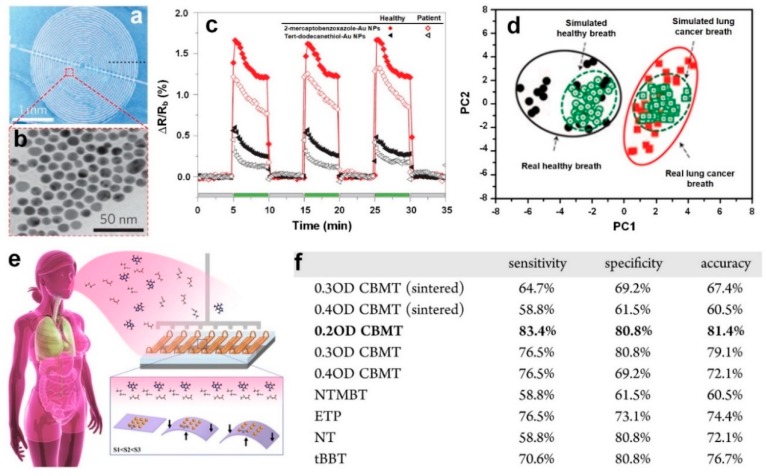
(**a**) SEM image of the device made of Au nanoparticles deposited on Si substrate with interdigitated gold electrodes. (**b**) TEM image of the deposited Au nanoparticles (dark dots) and the capping organic molecules (bright medium between the adjacent dark dots). (**c**) Sensing response of 2-mercaptobenzoxazole–gold nanoparticles (red diamonds) and *tert*-dodecanethiol–gold nano-particles (black triangles) upon exposure to headspace of healthy breath (filled symbols) and lung cancer breath (open symbols), as representative examples for sensors having negative responses. (**d**) PCA of the dataset of real and simulated human breath for lung cancer and healthy people [10]. Reproduced with permission [10], Copyright 2009, Springer Nature. (**e**) Schematic illustration of sensing the fabricated device consists of multiple sensors exposed to human breath. The sensors are strained in multiple bending steps leading to unique responses to the targeting VOCs. (**f**) Single sensor analysis results, providing the separation ability of each individual sensor [56]. Reproduced with permission [56], Copyright 2015, ACS Publications.

The sensing results of the fabricated arrays of sensors were investigated by principal component analysis (PCA) to evaluate the chemiresistive electronic nose selectivity. The result highlighted a distinguishable detection of the targeted gases NH_3_, NO, and H_2_S (Figure 7m), using both the Au-functionalized thin films and the VLNs [18]. This excellent sensing performance in distinguishing chemical gases in an 80% RH and relatively low temperature demonstrated the fabricated arrays of sensors as a promising candidate for monitoring health problems such as kidney disorders and asthma using human breath [18]. By combining the benefits of organic specificity with inorganic chemiresistive materials, Peng et al. [10] fabricated an array of nine sensors made of gold nanoparticles, capable of rapidly distinguishing the breath of lung cancer patients from healthy individuals in RH of 80%. The sensors were fabricated by depositing 5 nm gold nanoparticles on interdigitated gold electrodes (Figure 8a,b) which were subsequently functionalized by different organic materials including decanethiol, dodecanethiol, 2-ethylhexanethiol and 1-butanethiol [10]. In the first phase of their work, they identified 42 VOCs in exhaled breath representing lung cancer biomarkers, and later, they used four of these VOCs (~145 ppb ethylbenzene, ~67 ppb 4-methyl-octane, ~24 ppb undecane, and ~20 ppb 2,3,4-trimethyl-hexane) to optimize their fabricated sensor arrays [10]. Similar to previous research [18], the sensors were tested individually and their response to the target biomarkers were firstly examined, resulting in a rapid and fully reversible response (Figure 8c) to a wide variety of biomarker concentrations, with a detection limit of 1–5 ppb [10]. Later, the obtained response of the array of sensors was analyzed using the PCA method resulting in a clear discrimination with no overlap between the healthy and lung cancer patterns (Figure 8d) [10]. As it is presented in Figure 8d, no overlaps were observed between the simulated lung cancer breath and healthy breath mixtures, using the fabricated gold nanoparticles based nine-sensor arrays. The simulated data was produced from sampling of real exhaled breath samples by GC-MS. A mixture of representative VOCs was then produced by a computer-controlled automated flow system having a similar composition to those extracted earlier by the GC-MS. Although smaller in both cases the clusters of simulated breath samples strongly correlate with and separate both the real lung cancer and healthy breath samples indicating the robustness of the simulation approach as well as justification of the choice for the four out of the 42 most influential identified biomarkers [10].

In a similar approach, Kahn et al. [56] fabricated a flexible array of sensors based on molecularly modified gold nanoparticles (Figure 8e), capable of selectively detecting ppb level VOCs representative of ovarian cancer in exhaled breath, with up to 82% accuracy. An array of 10 sensors was fabricated using different ligands, film thicknesses and sintering times, and then exposed to seven VOCs representative of ovarian cancer, while recording the sensors’ electrical resistance. Among different ligands, chlorobenzenemethanethiol (CBMT) demonstrated a significantly higher selectivity, and thus, was further evaluated to identify the optimal film thickness optical density (OD) [56]. Figure 8f presents the results obtained from analyzing a single sensor providing the sensors sensitivity, specificity and accuracy. It is observed that a 0.2 OD CBMT sensor alone could provide comparable separation with that obtained by utilizing an extensive variety of several sensors [56]. A promising proof of concept that could eventually develop into a system capable of extracting the data required for real breath analysis.

## 6. Summary and Outlook

Non-invasive detection of diseases by analyzing human breath is a fast, low-cost and a simple alternative to blood analysis. Using semiconducting metal oxide gas sensors capable of analyzing human breath for medical applications have recently attracted great attention because of their high sensitivity, simple device fabrication, and great miniaturization possibility. Despite several challenges such as sensor’s selectivity, slow dynamic response and high operating temperature significant effort and careful investigation of different types of metal oxide semiconductor-based sensors including doped, composite and p-n heterojunction devices is proceeding with a focus on identifying the optimum nanostructure architecture and morphology. Regardless chemiresistive sensors based on metal oxide semiconductors are showing promising early results in the diagnostics of numerous diseases such as lung and breast cancers, asthma and diabetes. However, comprehensive work remains to be carried out regarding current and future technologies for diseases diagnosis using chemiresistive gas sensors.
